# The Hidden Diversity of *Zanclea* Associated with Scleractinians Revealed by Molecular Data

**DOI:** 10.1371/journal.pone.0133084

**Published:** 2015-07-24

**Authors:** Simone Montano, Davide Maggioni, Roberto Arrigoni, Davide Seveso, Stefania Puce, Paolo Galli

**Affiliations:** 1 Department of Biotechnologies and Biosciences, University of Milan-Bicocca, Piazza della Scienza 2, 20126, Milan, Italy; 2 MaRHE Center (Marine Research and High Education Center), Magoodhoo Island, Faafu Atoll, Republic of Maldives; 3 Red Sea Research Center, Division of Biological and Environmental Science and Engineering, King Abdullah University of Science and Technology, Thuwal, 23955-6900, Saudi Arabia; 4 Department of Life and Environmental Sciences, Polytechnic University of Marche, Via Brecce Bianche, 60131, Ancona, Italy; Laboratoire Arago, FRANCE

## Abstract

Scleractinian reef corals have recently been acknowledged as the most numerous host group found in association with hydroids belonging to the *Zanclea* genus. However, knowledge of the molecular phylogenetic relationships among *Zanclea* species associated with scleractinians is just beginning. This study, using the nuclear 28S rDNA region and the fast-evolving mitochondrial 16S rRNA and COI genes, provides the most comprehensive phylogenetic reconstruction of the genus *Zanclea* with a particular focus on the genetic diversity among *Zanclea* specimens associated with 13 scleractinian genera. The monophyly of *Zanclea* associated with scleractinians was strongly supported in all nuclear and mitochondrial phylogenetic reconstructions. Furthermore, a combined mitochondrial 16S and COI phylogenetic tree revealed a multitude of hidden molecular lineages within this group (Clades I, II, III, V, VI, VII, and VIII), suggesting the existence of both host-generalist and genus-specific lineages of *Zanclea* associated with scleractinians. In addition to *Z*. *gallii* living in association with the genus *Acropora*, we discovered four well-supported lineages (Clades I, II, III, and VII), each one forming a strict association with a single scleractinian genus, including sequences of *Zanclea* associated with *Montipora* from two geographically separated areas (Maldives and Taiwan). Two host-generalist *Zanclea* lineages were also observed, and one of them was formed by *Zanclea* specimens symbiotic with seven scleractinian genera (Clade VIII). We also found that the COI gene allows the recognition of separated hidden lineages in agreement with the commonly recommended mitochondrial 16S as a DNA barcoding gene for Hydrozoa and shows reasonable potential for phylogenetic and evolutionary analyses in the genus *Zanclea*. Finally, as no DNA sequences are available for the majority of the nominal *Zanclea* species known, we note that they will be necessary to elucidate the diversity of the *Zanclea*-scleractinian association.

## Introduction

Hydroids belonging to the genus *Zanclea* Gegenbaur, 1857 (Cnidaria, Hydrozoa) are distributed worldwide [1–5] and can be found from the intertidal zone [6–8] up to a depth of 500 m [9]. Of all 34 nominal species ascribed to this genus, a dozen have been described exclusively based on medusa specimens collected using plankton nets [10–14]. The remaining *Zanclea* species, identified through observation of both polyp and medusa stages, are known to have a preference for living substrates, usually forming symbiotic relationships with marine organisms such as bivalves, octocorals and bryozoans [5, 15–20]. Scleractinian reef corals are traditionally known to host many taxa of associated organisms [21, 22]; recently, several studies have revealed that the genus *Zanclea* is an additional component of this plethora of symbioses [6–8, 15].

After a few restricted preliminary reports from Mozambique [23, 24] and Papua New Guinea [15], an increasing number of studies on *Zanclea*-scleractinian symbiosis have recently been published focusing on different aspects of this close association such as ecology, taxonomy, physical interactions, and geographical distribution [6–8, 25–29]. The association with scleractinians currently involves the four species *Zanclea gilii* Boero, Bouillon & Gravili, 2000; *Zanclea margaritae* Pantos & Bythell, 2010; *Zanclea sango* Hirose & Hirose, 2011; and *Zanclea gallii* Montano, Maggioni & Puce 2014 and some as yet unidentified species [6–8, 15, 26–30]. All those species belong to the “*polymorpha* group” showing colonies of hydroids consisting of both retractile gastro-gonozooids and dactylozooids [15]. The geographic distribution of this association includes the Red Sea [27] and several Indo-Pacific regions such as Australia, Indonesia, Taiwan, Japan and the Republic of Maldives [7, 8, 26, 30]. The host range currently includes approximately 24 scleractinian genera belonging to 7 families, with a total of 33 scleractinian species involved [29]. Thus, reef-building corals are the host group with the highest number of species found in association with *Zanclea* species.

Fontana et al. [26] recently proposed a genus-specific association between *Zanclea* and scleractinians. However, whereas *Z*. *gallii*, *Z*. *margaritae*, and the unidentified *Zanclea* specimens studied by Fontana et al. [26] settle locally on genus *Acropora* [6, 28], *Z*. *sango* is a more generalist species living on the genera *Pavona* and *Psammocora* and it shows a widespread distribution [28]. Unfortunately, except for these preliminary data, no other information at the species level is available regarding the host-specificity and diversity of *Zanclea* associated with scleractinians. Differences in the hydroid colony, the absence and presence of perisarc and the cnidome of both the polyp and medusa stages are the morphological features generally used to identify *Zanclea* species [7, 15, 16, 19, 28, 31]. Considering that the diversity of this genus, as well as of many cnidarians, could be underestimated due to the difficulty of morphologic identification, molecular techniques, as part of an ‘integrated taxonomy’ approach [32], may be very useful.

Knowledge regarding the molecular phylogenetic relationships among *Zanclea* species associated with scleractinians is still far from complete. In fact, with the exception of the recent description of *Z*. *gallii* based on an integrated morpho-molecular approach [28], the other three *Zanclea* species have been described only through the study of their morphological characters [6, 7, 15]. At present, mitochondrial and nuclear phylogenetic analyses have shown that all the available sequences of *Zanclea* associated with scleractinians form a monophyletic lineage clearly separated from the genus type species *Zanclea costata* Gegenbaur, 1857 [26, 28]. Within this cohesive group, both *Z*. *sango* and *Z*. *gallii* were recovered as distinct monophyletic lineages based on partial 16S gene sequences, with the latter species closely related but molecularly separated from the unidentified *Acropora*-associated *Zanclea* specimens studied by Fontana et al. [26, 28]. However, no sequences are currently available for *Z*. *gilii* and *Z*. *margaritae*.

The mitochondrial cytochrome c oxidase I (COI) gene has been broadly adopted as a barcoding gene for animal life [33, 34]. Nevertheless, its utility has been strongly criticized in some animals at the base of the Metazoan tree, such as Porifera and Cnidaria, due to the slow nucleotide substitution rate of the mitochondrial genome resulting in an overlap between intra- and interspecific divergence [35–37]. Concerning Hydrozoa, although in some cases this gene has been revealed as phylogenetically informative [38–40], the mitochondrial 16S rRNA gene has been preferentially used being highly variable, easy to amplify and useful for distinguishing nominal and cryptic hydroid species [28, 41–46]. For these reasons, the mitochondrial 16S gene has been proposed as a barcode across Hydrozoa [45].

Herein, we collected 63 specimens of *Zanclea* living on 13 scleractinian genera in Faafu Atoll, Maldives, which represents an area hosting a relatively high number of reef coral genera currently known to be involved in this symbiosis [29]. The genetic diversity and the phylogenetic relationships of *Z*. *sango*, *Z*. *gallii*, and several other unidentified *Zanclea* specimens associated with different scleractinian hosts were investigated by sequencing three molecular markers, the nuclear 28S rDNA region and the fast-evolving mitochondrial genes, 16S rRNA and COI genes, to evaluate the molecular diversity and degree of host specificity of *Zanclea* associated with scleractinians. Furthermore, we evaluated whether the COI gene is phylogenetically informative and appropriate among *Zanclea* species associated with scleractinians.

## Material and Methods

### Sample collection

**Fig 1:**
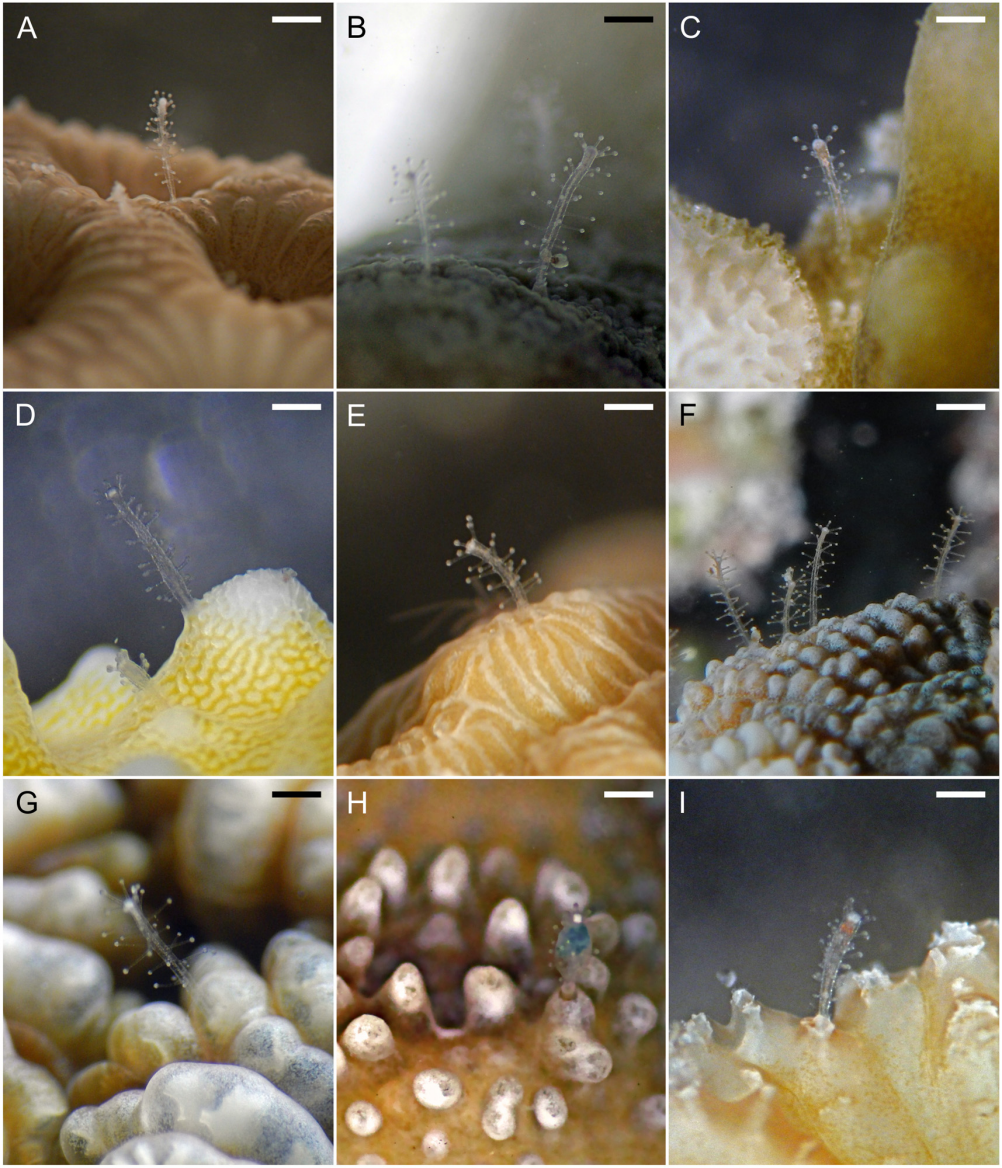
*In situ* photographs and microphotographs of living *Zanclea* hydroids associated with scleractinians. **A**) *Goniastrea*; **B**) *Porites*; **C**) *Montipora*; **D**) *Acropora*; **E**) *Pavona*; **F**) *Favites*; **G**) *Dipsastrea*; **H**) *Echinopora*; **I**) *Platygyra*. (Scale bars: ~ 500 μm)

The sampling was conducted between March and May 2014 in the waters around Magoodhoo Island, Faafu Atoll, Republic of Maldives (3°04’ N; 72°57’ E) (S1 Fig). The presence of *Zanclea* on scleractinian genera was recorded qualitatively *in situ*. Up to 13 scleractinian genera hosting *Zanclea* were selected and small fragments were collected for each of them. Single hydroid polyps were carefully collected one by one using a syringe needle directly from a bowl filled with seawater placed under a stereomicroscope. Afterwards, they were immediately preserved in 95% ethanol for further molecular analyses and fixed in 4% formalin for taxonomic identification. For documentary purposes we took underwater photographs of *Zanclea*-coral associations using a Canon G11 camera in a Canon WP-DC 34 underwater housing (Fig 1). Microphotographs (32x) of hydroids protruding from the coral skeletons were taken by use of a Leica EZ4 D stereomicroscope equipped with a Canon G11 camera (Fig 1). All hydroids (except *Z*. *gallii* and *Z*. *sango*) were identified at genus level according to Bouillon et al. [47], while the scleractinian hosts were identified to genus level according to updated taxonomic classifications: Acroporidae [48, 49], Agariciidae [50], Dendrophylliidae [51, 52], Lobophylliidae [53, 54], Merulinidae [53, 55, 56], Poritidae [57].

### Ethics Statement

The field study was approved by the Ministry of Fisheries and Agriculture of the Republic of Maldives and it did not involve endangered or protected species.

### Molecular analyses

The total genomic DNA of 63 ethanol-fixed *Zanclea* samples from 13 scleractinian genera was extracted following a protocol modified from Zietara et al. [58]. Three different molecular markers were amplified: (1) a ~300 bp portion of the nuclear 28S ribosomal DNA gene (28S), (2) a ~400 bp portion of the mitochondrial 16S ribosomal RNA gene (16S), and (3) a ~700 bp portion of the mitochondrial cytochrome oxidase subunit I gene (COI). The first two regions of DNA have been extensively used to infer phylogenetic relationships among hydroids in numerous previous molecular studies [26, 28, 44, 45, 59–61]. We also selected the barcoding region of COI gene because it turned out to be useful for species delimitation in Hydrozoa [40, 62]. 16S and 28S genes were amplified using hydroid-specific primers and the protocols proposed by Fontana et al. [26]. The barcoding region of COI gene was amplified using universal primers LCO1490 and HCO2198 and the protocol proposed by Folmer et al. [63]. All PCR products were purified and directly sequenced in forward and reverse directions using an ABI 3730xl DNA Analyzer (Applied Biosystem, Foster City, CA, USA). The sequences obtained in this study were deposited with the EMBL, and the accession numbers are listed in Table 1.

**Table 1:** List of specimens. List of specimens included in the analysis, with specimen code, locality and GenBank accession numbers, when available.

				Genbank accession numbers		
Species	Coral host genus	Specimen code	Locality	COI	16S	28S
*Zanclea gallii*	*Acropora*	MA056	Maldives	**LN714228**	**LN714105**	**LN714169**
*Zanclea gallii*	*Acropora*	MA057	Maldives	**LN714229**	**LN714106**	**LN714170**
*Zanclea gallii*	*Acropora*	MA058	Maldives		**LN714107**	**LN714171**
*Zanclea gallii*	*Acropora*	MA059	Maldives	**LN714230**	**LN714108**	**LN714172**
*Zanclea gallii*	*Acropora*	AC1	Maldives	**LN794213**	LK934472	LK934479
*Zanclea sango*	*Pavona*	MA051	Maldives	**LN714225**	**LN714100**	**LN714164**
*Zanclea sango*	*Pavona*	MA052	Maldives	**LN714226**	**LN714101**	**LN714165**
*Zanclea sango*	*Pavona*	MA053	Maldives	**LN714227**	**LN714102**	**LN714166**
*Zanclea sango*	*Pavona*	MA054	Maldives		**LN714103**	**LN714167**
*Zanclea sango*	*Pavona*	PA1	Maldives		LK934475	LK934483
*Zanclea* sp.	*Goniastrea*	MA001	Maldives	**LN714179**	**LN714050**	**LN714115**
*Zanclea* sp.	*Goniastrea*	MA002	Maldives	**LN714180**	**LN714051**	**LN714116**
*Zanclea* sp.	*Goniastrea*	MA003	Maldives	**LN714181**	**LN714052**	**LN714117**
*Zanclea* sp.	*Goniastrea*	MA004	Maldives	**LN714182**	**LN714053**	**LN714118**
*Zanclea* sp.	*Goniastrea*	MA005	Maldives	**LN714183**	**LN714054**	**LN714119**
*Zanclea* sp.	*Favites*	MA006	Maldives	**LN714184**	**LN714055**	**LN714120**
*Zanclea* sp.	*Favites*	MA007	Maldives	**LN714185**	**LN714056**	**LN714121**
*Zanclea* sp.	*Favites*	MA008	Maldives	**LN714186**	**LN714057**	**LN714122**
*Zanclea* sp.	*Favites*	MA009	Maldives	**LN714187**	**LN714058**	**LN714123**
*Zanclea* sp.	*Favites*	MA010	Maldives	**LN714188**	**LN714059**	**LN714124**
*Zanclea* sp.	*Dipsastrea*	MA011	Maldives	**LN714189**	**LN714060**	**LN714125**
*Zanclea* sp.	*Dipsastrea*	MA012	Maldives	**LN714190**	**LN714061**	**LN714126**
*Zanclea* sp.	*Dipsastrea*	MA013	Maldives	**LN714191**	**LN714062**	**LN714127**
*Zanclea* sp.	*Dipsastrea*	MA014	Maldives		**LN714063**	
*Zanclea* sp.	*Dipsastrea*	MA015	Maldives		**LN714064**	**LN714128**
*Zanclea* sp.	*Leptoseris*	MA016	Maldives	**LN714192**	**LN714065**	**LN714129**
*Zanclea* sp.	*Leptoseris*	MA017	Maldives	**LN714193**	**LN714066**	**LN714130**
*Zanclea* sp.	*Leptoseris*	MA018	Maldives	**LN714194**	**LN714067**	**LN714131**
*Zanclea* sp.	*Leptoseris*	MA019	Maldives	**LN714195**	**LN714068**	**LN714132**
*Zanclea* sp.	*Leptoseris*	MA020	Maldives	**LN714196**	**LN714069**	**LN714133**
*Zanclea* sp.	*Leptastrea*	MA021	Maldives	**LN714197**	**LN714070**	**LN714134**
*Zanclea* sp.	*Leptastrea*	MA022	Maldives	**LN714198**	**LN714071**	**LN714135**
*Zanclea* sp.	*Leptastrea*	MA023	Maldives	**LN714199**	**LN714072**	**LN714136**
*Zanclea* sp.	*Leptastrea*	MA024	Maldives	**LN714200**	**LN714073**	**LN714137**
*Zanclea* sp.	*Leptastrea*	MA025	Maldives	**LN714201**	**LN714074**	**LN714138**
*Zanclea* sp.	*Echinopora*	MA026	Maldives		**LN714075**	**LN714139**
*Zanclea* sp.	*Echinopora*	MA027	Maldives	**LN714202**	**LN714076**	**LN714140**
*Zanclea* sp.	*Echinopora*	MA028	Maldives	**LN714203**	**LN714077**	**LN714141**
*Zanclea* sp.	*Echinopora*	MA029	Maldives		**LN714078**	**LN714142**
*Zanclea* sp.	*Echinopora*	MA030	Maldives	**LN714204**	**LN714079**	**LN714143**
*Zanclea* sp.	*Turbinaria*	MA031	Maldives	**LN714205**	**LN714080**	**LN714144**
*Zanclea* sp.	*Turbinaria*	MA032	Maldives	**LN714206**	**LN714081**	**LN714145**
*Zanclea* sp.	*Turbinaria*	MA033	Maldives	**LN714207**	**LN714082**	**LN714146**
*Zanclea* sp.	*Turbinaria*	MA034	Maldives	**LN714208**	**LN714083**	**LN714147**
*Zanclea* sp.	*Turbinaria*	MA035	Maldives	**LN714209**	**LN714084**	**LN714148**
*Zanclea* sp.	*Platygyra*	MA036	Maldives	**LN714210**	**LN714085**	**LN714149**
*Zanclea* sp.	*Platygyra*	MA037	Maldives	**LN714211**	**LN714086**	**LN714150**
*Zanclea* sp.	*Platygyra*	MA038	Maldives	**LN714212**	**LN714087**	**LN714151**
*Zanclea* sp.	*Platygyra*	MA039	Maldives	**LN714213**	**LN714088**	**LN714152**
*Zanclea* sp.	*Platygyra*	MA040	Maldives	**LN714214**	**LN714089**	**LN714153**
*Zanclea* sp.	*Symphyllia*	MA041	Maldives	**LN714215**	**LN714090**	**LN714154**
*Zanclea* sp.	*Symphyllia*	MA042	Maldives	**LN714216**	**LN714091**	**LN714155**
*Zanclea* sp.	*Symphyllia*	MA043	Maldives	**LN714217**	**LN714092**	**LN714156**
*Zanclea* sp.	*Symphyllia*	MA044	Maldives	**LN714218**	**LN714093**	**LN714157**
*Zanclea* sp.	*Symphyllia*	MA045	Maldives	**LN714219**	**LN714094**	**LN714158**
*Zanclea* sp.	*Porites*	MA046	Maldives	**LN714220**	**LN714095**	**LN714159**
*Zanclea* sp.	*Porites*	MA047	Maldives	**LN714221**	**LN714096**	**LN714160**
*Zanclea* sp.	*Porites*	MA048	Maldives	**LN714222**	**LN714097**	**LN714161**
*Zanclea* sp.	*Porites*	MA049	Maldives	**LN714223**	**LN714098**	**LN714162**
*Zanclea* sp.	*Porites*	MA050	Maldives	**LN714224**	**LN714099**	**LN714163**
*Zanclea* sp.	*Montipora*	MA061	Maldives	**LN714232**	**LN714110**	**LN714174**
*Zanclea* sp.	*Montipora*	MA062	Maldives	**LN714233**	**LN714111**	**LN714175**
*Zanclea* sp.	*Montipora*	MA063	Maldives	**LN714234**	**LN714112**	**LN714176**
*Zanclea* sp.	*Montipora*	MA064	Maldives	**LN714235**	**LN714113**	**LN714177**
*Zanclea* sp.	*Montipora*	MA065	Maldives	**LN714236**	**LN714114**	**LN714178**
*Zanclea* sp.		XMZS1	China	KF962188	KF962532	KF962373
*Zanclea* sp.		XMZS2	China	KF962189	KF962533	KF962374
*Zanclea* sp.		XMZS3	China	KF962190	KF962534	KF962375
*Zanclea* sp.		XMZS4	China	KF962191	KF962535	KF962376
*Zanclea* sp.		XMZS5	China	KF962192	KF962536	KF962377
*Zanclea* sp.		XMZS6	China	KF962193	KF962537	KF962378
*Zanclea* sp.		XMZS7	China	KF962194	KF962538	KF962379
*Zanclea* sp.		XMZS8	China	KF962195	KF962539	KF962380
*Zanclea* sp.		XMZS9	China	KF962196	KF962540	KF962381
*Zanclea* sp.		XMZS10	China	KF962197	KF962541	KF962382
*Zanclea costata*		MHNG INV26507	France		EU876553	EU879951
*Zanclea costata*		MHNG INV26507	France		FN687559	
*Zanclea costata*			France		AY512531	
*Zanclea giancarloi*			France		FN687560	
*Zanclea giancarloi*			France		FN687561	
*Zanclea giancarloi*			Spain		FN687562	
*Zanclea sessilis*			Spain		AY512532	
*Zanclea sessilis*		MHNG INV61438	France		FN687557	
*Zanclea sessilis*			Spain		FN687558	
*Zanclea prolifera*		KUNHM 2793	Japan		EU305488	EU272598
*Asyncoryne ryniensis*		KUNHM 2639	Japan		EU876552	GQ424289
*Cladocoryne floccosa*					EU876554	EU272551
*Hydra vulgaris*			Argentina			EU879941
*Hydrocoryne miurensis*		KUNHM 2814	Japan			GQ424313
*Millepora* sp.					EU876551	EU879950
*Moerisia* sp.			California		AY512534	AY920801
*Moerisia* sp.					EU876555	
*Odessia maeotica*		MHNG INV53642	France		GQ395324	GQ424314
*Olindias sambaquiensis*			Brazil		EU293977	
*Pennaria disticha*		MHNG INV29809	Spain			GQ424316
*Porpita porpita*					AY935322	EU883551
*Porpita* sp.			Guam			AY920803
*Solanderia ericopsis*			New Zealand		AY512530	
*Solanderia ericopsis*		MHNG INV29593	New Zealand		AY787881	EU272593
*Solanderia secunda*		KUNHM 2611	Japan		EU305484	EU305533
*Sphaerocoryne agassizii*			Florida		GQ395323	GQ424318
*Stauridiosarsia cliffordi*		MHNG INV36025	Canada		GQ395313	
*Stauridiosarsia producta*		MHNG INV48751	Norway			GQ424301
*Velella* sp.		AGC1031				EU272597

**Boldface** indicates newly obtained sequences.

### Molecular phylogenetic analyses and haplotype network

The chromatograms were viewed, edited, and assembled using CodonCode Aligner 3.7.0 (CodonCode Corporation, Dedham, MA, USA). Alignments of the three separate datasets were generated using the E-INS-i option in MAFFT 7.110 [64, 65] with default parameters. Genetic distances (Kimura 2-parameter) within and among nominal *Zanclea* species and/or our *Zanclea* molecular lineages were computed for each separated molecular locus using MEGA 6 [66].

To examine whether the sequences from 16S and COI loci should be combined in a single analysis, a partition-homogeneity test was run in PAUP 4.0b1 [67], and significance was estimated by 1000 repartitions. This test, described as the incongruence-length divergence test by Farris et al. [68], indicated no conflicting phylogenetic signals between the datasets (*P* = 0.99). Therefore, 16S and COI were linked and datasets from both molecular markers were concatenated into a single data matrix, while the 28S sequences were considered as a separate set. Single 16S and COI trees are reported in S2 and S3 Figs, respectively. The newly obtained 28S sequences of *Zanclea* were aligned with other homologous ones available in GenBank and DRYAD databases (DOI: http://dx.doi.org/10.5061/dryad.g0b20) and belonging both to the genus *Zanclea* and to other families of the clade Capitata [26, 28, 59, 60, 69] (Table 1). *Hydra vulgaris*, a representative of the clade Aplanulata [59], was selected as outgroup due to its divergence from the clade Capitata [60, 70]. For the concatenated 16S and COI dataset, our newly obtained *Zanclea* sequences were aligned with homologous sequences of *Zanclea* sp. available in GenBank and coming from China Sea and unknown host (Table 1). We selected these sequences because of their sister relationship with our scleractinian-associated *Zanclea* sequences as shown in the 28S analyses. Phylogenetic analyses were performed using three methods: Maximum Parsimony (MP), Bayesian Inference (BI), and Maximum Likelihood (ML). MP analyses were performed using PAUP4.0b10 with heuristic searches stepwise addition and tree-bisection-reconnection (TBR) branch swapping. The node consistency was assessed using 500 bootstrap replicates with randomly added taxa. The software MrModeltest2.3 [71] was used, in conjunction with PAUP4.0b10, to select the best-fit nucleotide substitution models for each locus. The most suitable models estimated using the Akaike information criterion (AIC) were GTR + I + for 28S, HKI + I + for 16S, and GTR + I for COI. BI analyses were performed using MrBayes 3.1.2 [72]. Four parallel Markov Chain Monte Carlo runs (MCMC) were conducted for 5 x 10^7^ generations for 28S and COI loci, 6x 10^7^ generations for 16S locus, and 6 x 10^7^ for the combined 16S and COI loci. Trees were sampled every 100 generations for each analysis, and the initial 25% of the total trees were discarded as burn-in based on checking the parameter estimates and convergence using Tracer 1.5 [73]. ML trees were built with PhyML 3.0 [74] using the evolutionary models selected by MrModeltest2.3 and the robustness of each clade was tested using 500 bootstrap replications.

Finally, sequences were converted into the Roehl format using DnaSP 5 [75] and haplotype networks for separate 16S and COI datasets were constructed in Network 4.6.1.2 (http://www.fluxus-technology.com) using the median-joining algorithm [76] and default settings.

## Results

### Molecular results

The total genomic DNA of 63 ethanol-fixed *Zanclea* samples from 13 scleractinian genera was extracted, and three molecular markers were amplified (28S, 16S and COI) for a total number 183 sequences.

**Fig 2:**
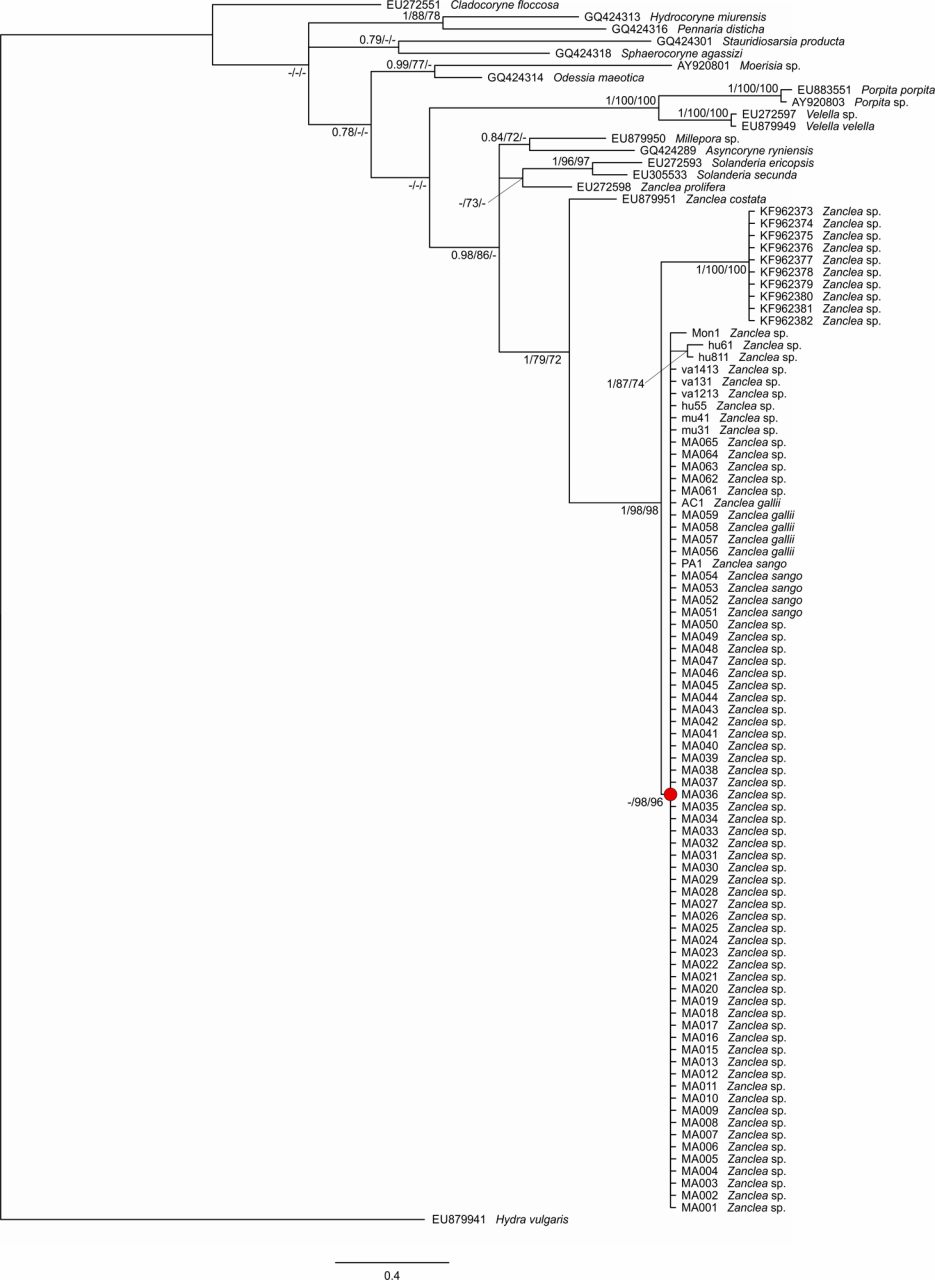
Phylogenetic tree based on the nuclear gene 28S inferred by Bayesian inference. The clade support values are *a posteriori* probabilities, bootstrap values from Maximum Likelihood, and bootstrap values from Maximum Parsimony, in this order. The node supporting the scleractinian-associated *Zanclea* clade is highlighted in red.

**Fig 3:**
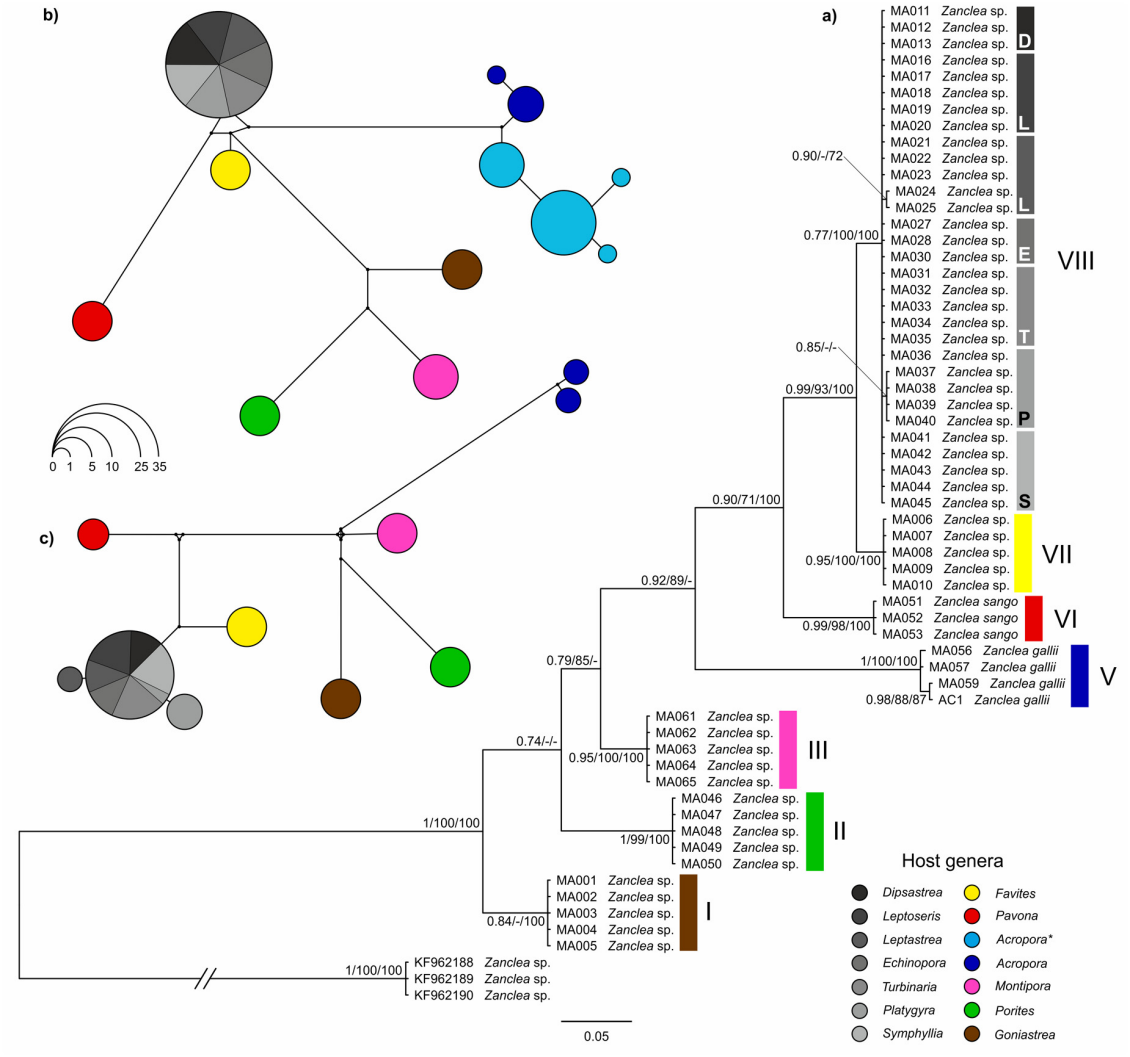
Phylogenetic trees and haplotype network analyses based on mithocondrial 16S and COI genes. **A**) Phylogenetic tree based on the combined mitochondrial genes 16S and COI inferred by Bayesian inference. The clade support values are *a posteriori* probabilities (≥ 0.7), bootstrap values from Maximum Likelihood (≥ 70), and bootstrap values from Maximum Parsimony (≥ 70), in this order. Clades of *Zanclea* associated with scleractinians are boxed in different colors depending on the host coral genera. **B-C**) Most parsimonious median-joining networks of *Zanclea* associated with scleractinians inferred from mitochondrial genes 16S (**B**) and COI (**C**). The size of circles is proportional to the frequencies of specimens sharing the same haplotype. The colors of circles referred to clades found in 3A. *Zanclea sp. sequences from Fontana et al. [26]

The total alignments of 28S, 16S, and COI datasets were respectively 252, 374, and 647 bp long, while the concatenated set of mitochondrial markers was 1009 bp long. Phylogenetic trees obtained from BI, ML, and MP analyses were similar and, therefore, only Bayesian topologies with significant branch support indicated by Bayesian posterior probability scores, ML bootstrapping supports, and MP bootstrapping supports were shown in Figs 2 and 3 and in S2 and S3 Figs.

The general topologies of 28S and 16S trees (Fig 2 and S2 Fig, respectively) were consistent with previous studies [26, 28]. They confirmed the paraphyly of the *Zanclea* genus, due to the divergent position of *Zanclea prolifera*. Furthermore, *Zanclea* associated with scleractinians and the other *Zanclea* species not living in association with hard coral are separated by high values of genetic distances, with a mean genetic distance of 6.1 ± 1.5% for 28S and 11.3 ± 1.4% for 16S. The monophyly of *Zanclea* associated with scleractinians was strongly supported in all the nuclear and mitochondrial phylogeny reconstructions. In the 28S analysis, all our newly obtained sequences clustered in a single lineage together with the other *Zanclea* associated with scleractinians sequences obtained from previous works [26, 28] but the relationships within this group were unresolved (Fig 2). 16S and COI trees were mostly congruent and their concatenation increased branch support values. Combined mitochondrial 16S and COI phylogenetic tree showed a better resolution of phylogenetic relationships among *Zanclea* associated with scleractinians and seven well-supported monophyletic lineages were identified (Clades I, II, III, V, VI, VII, and VIII) (Fig 3A, S2 and S3 Figs). 16S tree showed an additional clade (Clade IV) (S2 Fig), due to the presence in the analysis of *Acropora*-associated *Zanclea* sp. sequences from Fontana et al. [26], for which no COI sequences are currently available. Almost all of the seven *Zanclea* clades were genus-specific, except for Clade VIII that was associated with seven different host genera,. Hydroids belonging to Clade I were associated with *Goniastrea* and according to the concatenated analysis they represented the earliest diverging group of *Zanclea* associated with scleractinians (Fig 3A). Other early diverging clades were Clade II and Clade III, which included hydroids symbiotic respectively with *Porites* and *Montipora*. In the 16S tree, Clade III also included a specimen found on *Montipora* from Taiwan by Fontana et al. [26], for which there are no available COI data. *Acropora*-associated hydroids were monophyletic and themselves divided in two geographically distinct clades, Clade IV and Clade V, with the latter group corresponding to the nominal species *Z*. *gallii*. Clade VI was composed by hydroids belonging to the nominal species *Z*. *sango*, that we found in association with corals of the genus *Pavona*. Finally, *Zanclea* specimens of Clade VII were associated with *Favites*, while Clade VIII consisted of *Zanclea* samples found in association with *Dipsastrea*, *Echinopora*, *Leptastrea*, *Leptoseris*, *Platygyra*, *Symphyllia*, and *Turbinaria*. Within-clade genetic distances were extremely low for both mitochondrial markers being generally 0%, while inter-clade genetic distances were higher for COI rather than for 16S (Tables 2 and 3), with a mean of 6.9 ± 0.6% and 4.4 ± 0.7%, respectively. For example, the genetic distances between *Z*. *gallii* and *Z*. *sango* are 7.9 ± 1.1% for COI and 6.1 ± 1.4% for 16S (Tables 2 and 3).

**Table 2:** Pairwise comparisons and genetic distance. Pairwise comparisons of genetic distance within and between nominal species of *Zanclea* and/or clades of *Zanclea* associated with scleractinians based on the mitochondrial gene 16S.

Genetic distances	Clade I	Clade II	Clade III	Clade IV	Clade V	Clade VI	Clade VII	Clade VIII	*Zanclea* sp.*	*Z*. *giancarloi*	*Z*. *sessilis*	*Z*. *costata*	*Z*. *prolifera*
**Clade I**	0.0 (0.0)												
**Clade II**	3.2 (0.9)	0.0 (0.0)											
**Clade III**	2.9 (0.9)	2.6 (0.8)	0.0 (0.0)										
**Clade IV**	5.1 (1.2)	4.6 (1.1)	5.5 (1.2)	0.3 (0.2)									
**Clade V**	5.1 (1.3)	4.2 (1.1)	5.1 (1.2)	1.1 (0.5)	0.1 (0.1)								
**Clade VI**	4.1 (1.1)	5.7 (1.3)	5.4 (1.2)	6.5 (1.3)	6.1 (1.4)	0.0 (0.0)							
**Clade VII**	3.8 (1.1)	4.8 (1.1)	3.8 (1.0)	4.6 (1.1)	4.2 (1.1)	3.5 (1.0)	0.0 (0.0)						
**Clade VIII**	4.1 (1.1)	4.4 (1.1)	4.8 (1.1)	4.3 (1.1)	3.9 (1.1)	3.2 (1.0)	0.9 (0.5)	0.0 (0.0)					
***Zanclea* sp.** *	10.4 (1.8)	9.4 (1.7)	10.4 (1.8)	11.3 (1.9)	10.8 (1.9)	12.4 (2.0)	11.0 (1.8)	11.4 (1.8)	0.0 (0.0)				
***Z*. *giancarloi***	9.3 (1.6)	8.4 (1.5)	9.0 (1.6)	10.2 (1.7)	10.1 (1.7)	11.4 (1.8)	9.4 (1.6)	9.1 (1.5)	9.1 (1.6)	1.4 (0.5)			
***Z*. *sessilis***	9.4 (1.6)	8.3 (1.5)	8.6 (1.5)	10.8 (1.8)	10.7 (1.8)	11.3 (1.7)	9.9 (1.7)	9.6 (1.6)	9.4 (1.6)	5.7 (1.2)	1.0 (0.4)		
***Z*. *costata***	12.1 (1.7)	12.5 (1.7)	12.2 (1.7)	13.6 (1.9)	13.4 (1.9)	14.3 (2.0)	12.4 (1.8)	12.7 (1.8)	11.2 (1.7)	7.9 (1.3)	8.8 (1.4)	4.0 (0.9)	
***Z*. *prolifera***	15.2 (2.3)	15.2 (2.2)	16.6 (2.4)	18.0 (2.5)	17.4 (2.4)	17.3 (2.4)	16.2 (2.3)	16.6 (2.3)	15.5 (2.2)	14.1 (2.1)	15.6 (2.2)	15.7 (2.2)	n.c.

*Zanclea sp. sequences from China available in GenBank.

n.c. not calculated

Standard deviations are indicated in brackets.

**Table 3:** Pairwise comparisons and genetic distance. Pairwise comparisons of genetic distance within and between species of *Zanclea* and/or clades of *Zanclea* associated with scleractinians based on the mitochondrial gene COI.

	Clade I	Clade II	Clade III	Clade V	Clade VI	Clade VII	Clade VIII	*Zanclea* sp.*
**Clade I**	0.0 (0.0)							
**Clade II**	6.9 (1.0)	0.0 (0.0)						
**Clade III**	4.9 (0.9)	5.2 (0.9)	0.0 (0.0)					
**Clade V**	9.3 (1.2)	7.5 (1.1)	8.4 (1.2)	0.3 (0.2)				
**Clade VI**	8.5 (1.2)	7.6 (1.1)	7.3 (1.1)	7.9 (1.1)	0.0 (0.0)			
**Clade VII**	8.5 (1.2)	9.2 (1.3)	7.6 (1.2)	9.3 (1.2)	5.5 (0.9)	0.0 (0.0)		
**Clade VIII**	8.0 (1.1)	8.7 (1.2)	8.2 (1.2)	9.5 (1.3)	5.1 (0.9)	2.1 (0.6)	0.1 (0.0)	
***Zanclea* sp.** *	13.7 (1.4)	16.3 (1.7)	14.9(1.6)	16.7 (1.6)	13.7 (1.4)	16.2 (1.6)	15.4 (1.5)	0.0 (0.0)

*Zanclea sequences from China available in GenBank.

Standard deviations are indicated in brackets.

A total of 12 and 10 haplotypes were obtained respectively from 16S and COI sequences of *Zanclea* associated with scleractinians, Median-joining networks for each mitochondrial marker are shown in Fig 3B and 3C. Both networks were congruent with mitochondrial phylogenetic reconstructions and they are similar between each others. No haplotypes were shared between representatives of two or more clades identified with phylogenetic analyses and, thus, all of the clades were genetically separated from each other. COI haplotypes were separated by an approximate four times higher number of substitutions compared to 16S haplotypes. For example, the only two nominal species of *Zanclea* included in network analyses (*i*.*e*. *Z*. *gallii* and *Z*. *sango*) were separated by 26 substitutions in 16S network and by 81 substitution in COI network.

### Morphological results

For all the sampled hydroids, the morphology observed was in accordance with the description of the genus *Zanclea* [47]. The polyps arise abundantly from the scleractinian surface, being frequently scattered on the corallite edges or between corallites and have been recorded highly proximal to scleractinian polyps.

As already reported in Montano et al. [28], the morphological characters mainly used to distinguish *Zanclea* species are the organization of the colony (monomorphic or polymorphic), the presence of perisarc that covers the hydrorhiza and hydrocauli, the number of polyp tentacles, the placement of medusa buds on polyps, the cnidome of both polyps and medusae, and the number of cnidophores on the tentacles of medusae. The morphological characters of the clades resulted from the molecular analyses are reported in the Table 4 and in S4 Fig.

**Table 4:** Morphological differences among clades. Morphological characters of the clades resulted from the molecular analyses.

				Polyp tentacles		Cnidome		
Genetic clade	N° of host genera	Perisarc	Polymorphism	Oral	Aboral	Two-size stenoteles	Macrobasic euryteles	Medusae observation
I^a^	1	Yes	Unknown	6	25–36	Yes	Yes	No
II^a^	1	Unknown	Unknown	5–6	26–33	Yes	No	No
III^b^ ^,^ ^c^	1	Yes	Unknown	5–6	27–30	Yes	No	No
IV^b^	1	Unknown	Unknown	Unknown	Unknown	Unknown	Unknown	No
V^c^	1	No	Yes	4–6	14–26	Yes	No	Yes
VI^c^	2	Yes	Yes	4–6	11–22	Yes	Yes	Yes
VII^a^	1	Yes	Unknown	5–6	26–30	Yes	Yes	No
VIII^a^	7	Yes	Yes	5–7	23–35	Yes	Yes	No

^a^ present study;

^b^ Fontana et al. 2012;

^c^ Montano et al. 2015.

## Discussion

### *Zanclea* molecular phylogeny

The results provided in this study currently represent the most comprehensive phylogenetic reconstruction of the genus *Zanclea* with a particular focus on scleractinian-associated species. The broad-based phylogenetic trees obtained with both 28S and 16S molecular markers (Fig 2 and S2 Fig) are consistent with previous molecular studies [26, 60]. These trees essentially depict the genus *Zanclea* as a paraphyletic group within the Zancleida clade [26, 28, 60] due to the unresolved position of *Zanclea prolifera*. This species was formally classified in the genus *Zanclea* even though its polyp stage was unknown [11]. Furthermore, several molecular works have shown that *Z*. *prolifera* is more closely related to *Asyncoryne* spp. than to the other *Zanclea* species [26, 28, 60]. This genetic evidence is not unexpected considering that *Zanclea* and *Asyncoryne* have similar medusae [47, 77]. For this reason, several authors have proposed to move *Z*. *prolifera* into the genus *Asyncoryne* [15, 26, 60, 78], a hypothesis consistent with our 16S phylogenetic tree (S2 Fig).

Both the nuclear and mitochondrial phylogenetic reconstructions resolved *Zanclea* associated with scleractinians as a monophyletic lineage. As already discussed in Montano et al. [28], the monophyly of *Zanclea* associated with scleractinians is consistent with the recovery within the genus *Zanclea* of two distinct groups proposed by Boero et al. [15] mainly based on the occurrence of a monomorphic (the *alba* group) or polymorphic (the *polymorpha* group) colony. The latter group counts seven species to date, including three species associated with bryozoans (*Zanclea polymorpha* Schuchert, 1996, *Zanclea hirohitoi* Boero, Bouillon & Gravili 2000, and *Zanclea tipis* Puce, Cerrano, Boyer, Ferretti & Bavestrello, 2002) and the four currently described *Zanclea* species associated with scleractinians (*Z*. *gilii*, *Z*. *margaritae*, *Z*. *sango*, and *Z*. *gallii*). Therefore, the character state “polymorphic colony” could be consistent with the monophyly of *Zanclea* species associated with scleractinians and with their separation from *Zanclea* species showing a monomorphic colony. Nevertheless, detailed morphological data are not available for several specimens of *Zanclea* in symbiosis with scleractinians, and molecular data remain unavailable for most of the nominal species of *Zanclea*, including the polymorphic species associated with bryozoans. Therefore, the evolutionary validity of the distinction between the *alba* group and the *polymorpha* group needs to be further addressed in thefuture with full morphological and molecular analyses of *Zanclea* species ascribed to the two groups to undertake any formal taxonomic action.

### Genetic diversity of scleractinian-associated *Zanclea*

In addition to the commonly recommended mitochondrial 16S gene as a DNA barcode for Hydrozoa [40, 45, 79–81], we showed herein that the gene COI allows the recognition of separated hidden lineages in agreement with 16S data, revealing reasonable potential for phylogenetic and evolutionary analyses in the genus *Zanclea*. Indeed, COI turned out to be more variable than 16S, having approximately four times more mutations compared with 16S, despite the analysed portion of COI being bigger than that of 16S (647 bp for COI and 374 bp for 16S). Therefore, the levels of divergence observed within *Zanclea* associated with the scleractinian group strongly encourage and support the use of both COI and 16S sequences in phylogenetic studies of these hydroids. This conclusion is consistent also with several previous molecular works which successfully used COI gene in order to evaluate the potential presence of cryptic species or intraspecific population subdivision in *Plumularia setacea* [40], *Obelia geniculata* [38], and in the genus *Cordylophora* [81].

According to the mitochondrial phylogenetic trees and haplotype network analyses, all *Zanclea* specimens associated with scleractinians group together in a cohesive and monophyletic cluster; moreover, they are characterized by considerable genetic diversity (Fig 3A). Indeed, our molecular results indicate that this group is composed of multiple reciprocally well-supported monophyletic lineages (Clades I through VIII) that show a peculiar pattern of host specificity, as discussed in the following paragraph. Two of these seven lineages notably correspond to the nominal species *Z*. *sango* (Clade VI) and *Z*. *gallii* (Clade V), and the genetic divergence between the two species overlaps the distance values found between all the other molecular clades using both the mitochondrial 16S and COI genes (Tables 2 and 3). Although we are far from the establishment of an appropriate and widely accepted genetic distance threshold to differentiate hydrozoan species using 16S sequences, Moura et al. [45] proposed a conservative maximum of 2% divergence for intraspecific sequence distance in the Sertulariidae. In our 16S analysis, all the intraclade distances are under this value, while the interclade divergences exceed this conservative threshold in most of the pairwise comparisons. Furthermore the genetic differentiation of 16S locus between our multiple lineages of *Zanclea* (Table 2) is clearly consistent with those calculated between nominal and putative species of the genus *Turritopsis* (3.6%– 12.1%) [80] and *Acryptolaria* (up to 3.1%) [42]. Comparable 16S genetic distances revealed the existence of cryptic species within *Cordylophora* (3.3%- 6%) [81], *Nemertesia* (up to 4.8%) [43], *Stylactaria* (up to 6%) [46], *Cryptolaria pectinata* (up to 2.2%) [42], and *Lafoea dumosa* (up to 5%) [42, 44].

In conclusion, for both mitochondrial markers, relevant comparisons with previous similar works suggest that the genetic divergence found within *Zanclea* associated with scleractinians might be better explained by assigning independent species status to all molecular clades rather than considering these lineages to be the result of a strong population subdivision. Nevertheless, to discriminate between these two alternative hypotheses, it will be mandatory to corroborate our mitochondrial data with investigations of additional variable nuclear markers and to evaluate the possible presence of morphological features that are clade-diagnostic in the group of *Zanclea* associated with scleractinians.

### Host specificity of *Zanclea* associated with scleractinians

Currently, there is evidence concerning increasing reports of the occurrence of associations between scleractinians and hydroids belonging to the genus *Zanclea* in the coral community [6–8, 26, 27, 29]. This growing number of works likely reflects only a lack of attention about this association in previous decades, due to the small dimensions of hydroids, that have limited their observation. However, the absence of previous data prevents us from excluding a possible recent spread of this association in the reefs of the Indo-Pacific and the Red Sea. Furthermore, our molecular data showed that the genetic diversity within *Zanclea* associated with scleractinians is very high and that there is a multitude of hidden molecular lineages within this group. Boero et al. [15] hypothesized that radiation similar to bryozoan-inhabiting hydroids also occurred in coral-inhabiting hydroids, and the combined morpho-molecular data reported for the recently described species *Z*. *gallii* [28] as well as the molecular data obtained in the present study, seem to support this hypothesis.

With the exception of the less specialized *Z*. *alba* (Meyen, 1834), considered a species with characters near to the ancestral state, and *Z*. *costata*, which is not compulsorily associated with bivalves [15], the genus *Zanclea* usually shows high host specificity [15–19]. The present study suggests the existence of both host-generalist and genus-specific lineages of *Zanclea* associated with scleractinians. In addition to *Z*. *gallii* living in association with the genus *Acropora* in Maldives, we discovered four well-supported lineages (Clades I, II, III, and VII), each one forming a strict association with a single scleractinian genus. This evidence, together with the close relationship between sequences of *Zanclea* associated with *Montipora* from two geographically separated areas (Maldives and Taiwan), support the hypothesis that *Zanclea* in symbiosis with scleractinians include lineages that settle on scleractinian hosts belonging to a preferred genus, as already suggested by Fontana et al. [26]. However, two host-generalist *Zanclea* lineages were also observed. The first lineage includes *Z*. *sango*, a nominal species currently known to be associated with the two scleractinian genera *Pavona* and *Psammocora* [7, 28]. In addition, our analysis recovered a second well-supported lineage formed by *Zanclea* specimens symbiotic with seven scleractinian genera (Clade VIII). These two lineages could represent less specialized and more generalist *Zanclea* lineages living in association with several scleractinians ascribed to different genera.

Concerning morphological traits related to host specificity, Puce et al. [16] noted the importance of the presence or absence of a perisarc around the hydrorhiza. The authors suggested that ancestral species are predicted to be host generalists and characterized by hydrorhiza covered by a perisarc, whereas advanced species that establish specific associations with host species should have lost their perisarc. Although this scenario was already observed between *Z*. *gallii* and *Z*. *sango* [28], the morphological results herein obtained reveal the presence of a perisarc covering the hydrorhiza in both host-specific (Cades I, III and VII) and host-generalist (Clades VI and VIII) lineages. This evidence may suggest a less integrated relationship between *Zanclea* belonging to Clades I, III and VII and their host. An alternative hypothesis is that, as the presence of macrobasic euryteles [15], the absence of the perisarc, instead of being a derived character, might be due to independent events of loss and acquisition of the related structure. Despite the absence of some morphological information, the combined characters “perisarc” and “macrobasic euryteles” allow one to distinguish clades I, III and V. In addition, even though the presence of the perisarc is unknown, clade II differs from clade I, and in accord with the possible presence/absence of the perisarc it may be different from clade V or III, respectively. Clades I, VI, VII and VIII share the same state of the characters “perisarc” and “macrobasic euryteles”, but the last three represent a monophyletic clade and their similarities could be related to this condition. The character “polymorphic colony” was frequently unknown owing to the difficulty of noticing the presence of the very contractile dactylozooids. Three of the clades (V, VI, VIII) share polymorphic colonies, but additional investigations are required to determine whether this character is shared between all clades or if it may help to morphologically differentiate them. Moreover, knowledge of the life cycle of the specimens belonging to each clade will provide important information regarding the evolutionary history of *Zanclea* associated with scleractinians.

The available data prevent us from excluding the possibility that some *Zanclea* lineages, as some other cosmopolitan species of hydroids, may be complexes of species [82, 83]. Indeed, nominal species of hydroids known to have a very wide, circumglobal distribution could eventually result in different geographically delimited species [38, 46, 79, 81, 84, 85], sometimes suggesting the existence of cryptic species [40]. At present we can only speculate on the true diversity of *Zanclea* associated with scleractinians because the incomplete set of information currently available makes any discussion inconclusive. In fact, some *Zanclea* species lack complete morphological information, and no DNA sequences are available for the majority of the nominal *Zanclea* species known. Thus, we strongly stress that DNA sequences of already described *Zanclea* species are necessary to clarify the true diversity of the entire genus, and especially of species living in association with scleractinians.

## Conclusion

The recent literature [6–8, 26, 27, 29] suggests that the *Zanclea*-scleractinians symbiosis is widespread in coral communities of the Indo-Pacific and Red Sea. Although the analysis of species boundaries within the genus *Zanclea* is still far from complete, our results show that the barcoding region of the COI gene is very informative and useful in such scope. Herein, we set a starting point for further investigations, showing high genetic diversity in the *Zanclea*-scleractinian symbiosis and reporting potential hidden lineages both host-specific and host-generalist. Currently, the available morphological data suggest that some identified clades are morphologically different and that the possibility of crypticism between some molecular lineages is observed. Molecular phylogeny is currently revolutionizing the traditional systematics in a multitude of marine taxa including Hydrozoa [59, 60, 62]. Therefore, integration between a complete morphological approach that investigates both polyp and medusa stages and a molecular multilocus approach is needed to better clarify the diversity of the *Zanclea*-scleractinian association.

## Supporting Information

S1 Fig Map of the study area. A) Maldives; B) Faafu Atoll; C) Magoodhoo Island.
(TIF)

S2 Fig Phylogenetic tree based on the mitochondrial gene 16S inferred by Bayesian inference. The clade support values are *a posteriori* probabilities (≥ 0.7), bootstrap values from Maximum Likelihood (≥ 70), and bootstrap values from Maximum Parsimony (≥ 70), in this order.
(TIF)

S3 Fig Phylogenetic tree based on the mitochondrial gene COI inferred by Bayesian inference. The clade support values are *a posteriori* probabilities (≥ 0.7), bootstrap values from Maximum Likelihood (≥ 70), and bootstrap values from Maximum Parsimony (≥ 70), in this order.
(TIF)

S4 Fig Morphological characters of *Zanclea* hydroids associated with scleractinians. **A)** Gastrozooids and a dactylozooid (arrowhead) emerging from *Pavona varians*; **B-C)** Gastrogonozooid and a blastostyle bearing mature medusa buds on *Porites* sp. and *Acropora muricata*, respectively. **D)** An extended polyp belonging to clade VIII and growing on *Turbinaria* sp.; **E)** a contracted dactylozooid belonging to a *Zanclea sango* colony. **F-G)** Micrographs showing the basal portion of *Zanclea* hydroids associated with *Leptoseris* sp. and *Leptastrea* sp., respectively; the hydrocauli are covered by a transparent perisarc (arrowheads). **H)** Undischarged two-sized stenoteles; **I-J)** large and small discharged stenoteles. **K-L)** Undischarged apotrichous macrobasic eurytele from *Zanclea sango* and a detail of the distal part of the shaft of the same discharged nematocyst. (Scale bars: A-C ~ 0.5 mm; D-G ~ 100 μm; H-L ~ 5 μm).
(TIFF)

## References

[1] Kramp P (1968) The Hydromedusae of the Pacific and Indian Oceans. Sections II and III. Dana Rep 72: 1–200.

[2] Stepanjants S (1972) Hydroidea of the coastal waters of the Davis Sea (collected by the XIth Soviet Antarctic Expedition of 1965–1966). Biol res Soviet Antarct Exped 5: 56–79.

[3] Ristedt H and Schuhmacher H (1985) The bryozoan *Rhynchozoon larreyi* (Audouin, 1826)–A successful competitor in coral reef communities of the Red Sea. Mar Ecol 6: 167–179.

[4] Calder DR (1988) Shallow-Water Hydroids of Bermuda: The Athecatae. R Ont Mus Life Sci Contrib 148: 1–107.

[5] Gravili C, Boero F and Bouillon J (1996) *Zanclea* species (Hydroidomedusae, Anthomedusae) from the Mediterranean. Sci Mar 60: 99–108.

[6] Pantos O and Bythell JC (2010) A novel reef coral symbiosis. Coral Reefs 29: 761–770.

[7] Hirose M and Hirose E (2011) A new species of *Zanclea* (Cnidaria: Hydrozoa) associated with scleractinian corals from Okinawa, Japan. J Mar Biol Assoc U K 92: 877–884.

[8] Montano S, Maggioni D, Galli P, Seveso D and Puce S (2013) *Zanclea*–coral association new records from Maldives. Coral Reefs 32: 701.

[9] Bouillon J, Pagès F, Gili J-M, Palanques A, Puig P and Heussner S (2000) Deep-water Hydromedusae from the Lacaze-Duthiers submarine canyon (Banyuls, northwestern Mediterranean) and description of two new genera, *Guillea* and *Parateclaia* . Sci Mar 64: 87–95.

[10] Haeckel EHPA (1879) Monographie der Medusen. G. Fischer.

[11] Uchida T and Sugiura Y (1976) On a Hydromedusa, *Zanclea prolifera* n. sp., of which the medusa gives rise to medusa-buds. Proceedings of the Japan Academy 52: 141–144.

[12] Xu Z, Huang J and Chen X (1991) On new species and record of Hydromedusae in the upwelling region off the Minnan-Taiwan Bank fishing ground, China Minnan-Taiwan Bank Fishing Ground Upwelling Ecosystem Study (in Chinese) Beijing: Science Press 469: 486.

[13] Xu Z, Huang J and Guo D (2008) Six new species of Anthomedusae (Hydrozoa, Hydroidomedusae) from Beibu Gulf, China. In: Hu J. and Yang S., editors. Symposium on Oceanography of the Beibu Gulf I China Ocean Press, Beijing pp. 209–221.

[14] Gershwin LA and Zeidler W (2003) Encounter 2002 expedition to the isles of St Francis, South Australia: medusae, siphonophores and ctenophores. Trans R Soc S Aust 127: 205–241.

[15] Boero F, Bouillon J and Gravili C (2000) A survey of *Zanclea*, *Halocoryne* and *Zanclella* (Cnidaria, Hydrozoa, Anthomedusae, Zancleidae) with description of new species. Ital J Zool 67: 93–124.

[16] Puce S, Cerrano C, Boyer M, Ferretti C and Bavestrello G (2002) *Zanclea* (Cnidaria: Hydrozoa) species from Bunaken Marine Park (Sulawesi Sea, Indonesia). J Mar Biol Assoc U K 82: 943–954.

[17] Puce S, Bavestrello G, Di Camillo CG and Boero F (2007) Symbiotic relationships between hydroids and bryozoans. Symbiosis 44: 137–143.

[18] Puce S, Cerrano C, Di Camillo CG and Bavestrello G (2008) Hydroidomedusae (Cnidaria: Hydrozoa) symbiotic radiation. J Mar Biol Assoc U K 88: 1715–1721.

[19] Puce S, Di Camillo CG and Bavestrello G (2008) Hydroids symbiotic with octocorals from the Sulawesi Sea, Indonesia. J Mar Biol Assoc U K 88: 1643–1654.

[20] McKinney FK (2009) Bryozoan-hydroid symbiosis and a new ichnogenus, *Caupokeras* . Ichnos 16: 193–201.

[21] Stella JS, Pratchett MS, Hutchings PA and Jones GP (2011) Coral-associated invertebrates: diversity, ecological importance and vulnerability to disturbance. Oceanogr Mar Biol Annu Rev 49: 43–104.

[22] Hoeksema BW, Van der Meij SET and Fransen CHJM (2012) The mushroom coral as a habitat. J Mar Biol Assoc U K 92: 647–663.

[23] Millard NAH (1975) Monograph on the Hydroida of Southern Africa. Ann S Afr Mus 68: 1–513.

[24] Millard NAH and Bouillon J (1974) A collection of hydroids from Moçambique, East Africa. Ann S Afr Mus 65: 1–40.

[25] Pantos O and Hoegh-Guldberg O (2011) Shared skeletal support in a coral-hydroid symbiosis. PLoS One 6: e20946 10.1371/journal.pone.0020946 21695083 PMC3114865

[26] Fontana S, Keshavmurthy S, Hsieh HJ, Denis V, Kuo C-Y and Hsu C-M et al (2012) Molecular evidence shows low species diversity of coral-associated hydroids in *Acropora* corals. PLoS One 7: e50130 10.1371/journal.pone.0050130 23209655 PMC3510231

[27] Montano S, Galli P, Maggioni D, Seveso D and Puce S (2014) First record of coral-associated *Zanclea* (Hydrozoa, Zancleidae) from the Red Sea. Mar Biodivers 44: 581–584.

[28] Montano S, Arrigoni R, Pica D, Maggioni D and Puce S (2015) New insights into the symbiosis between *Zanclea* (Cnidaria, Hydrozoa) and scleractinians. Zool Scr 44: 92–105.

[29] Montano S, Seveso D, Galli P, Puce S and Hoeksema BW (2015) Mushroom corals as newly recorded hosts of the hydrozoan symbiont *Zanclea* sp. Mar Biol Res, 10.1080/17451000.2015.1009467

[30] Gravier-Bonnet N and Bourmaud C (2012) Hydroids (Cnidaria, Hydrozoa) of Baa Atoll (Indian Ocean, Maldives Archipelago). Atoll Res Bull: 82–123.

[31] Bouillon J, Medel MD, Pagès F, Gili JM, Boero F and Gravili C (2004) Fauna of the Mediterranean Hydrozoa. Sci Mar 68: 5–438.

[32] Dayrat B (2005) Towards integrative taxonomy. Biol J Linn Soc 85: 407–415.

[33] Hebert PD, Penton EH, Burns JM, Janzen DH and Hallwachs W (2004) Ten species in one: DNA barcoding reveals cryptic species in the neotropical skipper butterfly *Astraptes fulgerator* . Proc Natl Acad Sci U S A 101: 14812–14817. 15465915 10.1073/pnas.0406166101 PMC522015

[34] Hebert PD, Ratnasingham S and deWaard JR (2003) Barcoding animal life: cytochrome *c* oxidase subunit 1 divergences among closely related species. Proc Biol Sci 270 Suppl 1: S96–99. 12952648 10.1098/rsbl.2003.0025 PMC1698023

[35] Hellberg ME (2006) No variation and low synonymous substitution rates in coral mtDNA despite high nuclear variation. BMC Evol Biol 6: 24 16542456 10.1186/1471-2148-6-24 PMC1431588

[36] Shearer TL and Coffroth MA (2008) Barcoding corals: limited by interspecific divergence, not intraspecific variation. Mol Ecol Resour 8: 247–255. 10.1111/j.1471-8286.2007.01996.x 21585766

[37] Huang D, Meier R, Todd PA and Chou LM (2008) Slow mitochondrial COI sequence evolution at the base of the metazoan tree and its implications for DNA barcoding. J Mol Evol 66: 167–174. 10.1007/s00239-008-9069-5 18259800

[38] Govindarajan AF, Halanych KM and Cunningham CW (2005) Mitochondrial evolution and phylogeography in the hydrozoan *Obelia geniculata* (Cnidaria). Mar Biol 146: 213–222.

[39] Ortman BD, Bucklin A, Pagès F and Youngbluth M (2010) DNA Barcoding the Medusozoa using mtCOI. Deep-Sea Res Part II Top Stud Oceanogr 57: 2148–2156.

[40] Schuchert P (2014) High genetic diversity in the hydroid *Plumularia setacea*: a multitude of cryptic species or extensive population subdivision? Mol Phylogenet Evol 76: 1–9. 10.1016/j.ympev.2014.02.020 24602986

[41] Schuchert P and Reiswig HM (2006) *Brinckmannia hexactinellidophila*, n. gen., n. sp.: a hydroid living in tissues of glass sponges of the reefs, fjords, and seamounts of Pacific Canada and Alaska. Can J Zool 84: 564–572.

[42] Moura CJ, Cunha MR, Porteiro FM and Rogers AD (2012) Polyphyly and cryptic diversity in the hydrozoan families Lafoeidae and Hebellidae (Cnidaria: Hydrozoa). Invertebr Syst 25: 454.

[43] Moura CJ, Cunha MR, Porteiro FM, Yesson C and Rogers AD (2012) Evolution of *Nemertesia* hydroids (Cnidaria: Hydrozoa, Plumulariidae) from the shallow and deep waters of the NE Atlantic and western Mediterranean. Zool Scr 41: 79–96.

[44] Moura CJ, Harris DJ, Cunha MR and Rogers AD (2008) DNA barcoding reveals cryptic diversity in marine hydroids (Cnidaria, Hydrozoa) from coastal and deep-sea environments. Zool Scr 37: 93–108.

[45] Moura CJ, Cunha MR, Porteiro FM and Rogers AD (2011) The use of the DNA barcode gene 16S mRNA for the clarification of taxonomic problems within the family Sertulariidae (Cnidaria, Hydrozoa). Zool Scr 40: 520–537.

[46] Miglietta MP, Schuchert P and Cunningham CW (2009) Reconciling genealogical and morphological species in a worldwide study of the Family Hydractiniidae (Cnidaria, Hydrozoa). Zool Scr 38: 403–430.

[47] Bouillon J, Gravili C, Gili J-M and Boero F (2006) An introduction to Hydrozoa. Paris: Publications Scientifiques du Museum.

[48] Wallace CC, Done BJ and Muir PR (2012) Revision and Catalogue of Worldwide Staghorn Corals *Acropora* and *Isopora* (Scleractina: Acroporidae) in the Museum of Tropical Queensland. Mem Queensl Mus 57: 1–255.

[49] Wallace CC, Chen CA, Fukami H and Muir PR (2007) Recognition of separate genera within *Acropora* based on new morphological, reproductive and genetic evidence from *Acropora togianensis*, and elevation of the subgenus *Isopora* Studer, 1878 to genus (Scleractinia: Astrocoeniidae; Acroporidae). Coral Reefs 26: 231–239.

[50] Veron J (2000) Corals of the World Townsville, Australia: Australian Institute of Marine Science.

[51] Cairns SD (2001) A generic revision and phylogenetic analysis of the Dendrophylliidae (Cnidaria: Scleractinia). Smithson Contrib Zool 615: 1–75.

[52] Arrigoni R, Kitano YF, Stolarski J, Hoeksema BW, Fukami H, Stefani F, et al (2014) A phylogeny reconstruction of the Dendrophylliidae (Cnidaria, Scleractinia) based on molecular and micromorphological criteria, and its ecological implications. Zool Scr 43: 661–688.

[53] Budd AF, Fukami H, Smith ND and Knowlton N (2012) Taxonomic classification of the reef coral family Mussidae (Cnidaria: Anthozoa: Scleractinia). Zool J Linn Soc 166: 465–529.

[54] Arrigoni R, Terraneo TI, Galli P and Benzoni F (2014) Lobophylliidae (Cnidaria, Scleractinia) reshuffled: pervasive non-monophyly at genus level. Mol Phylogenet Evol 73: 60–64. 10.1016/j.ympev.2014.01.010 24472672

[55] Huang D, Benzoni F, Arrigoni R, Baird AH, Berumen ML, Bouwmeester J, et al (2014) Towards a phylogenetic classification of reef corals: the Indo-Pacific genera *Merulina*, *Goniastrea* and *Scapophyllia* (Scleractinia, Merulinidae). Zool Scr 43: 531–548.

[56] Huang D, Benzoni F, Fukami H, Knowlton N, Smith ND and Budd AF (2014) Taxonomic classification of the reef coral families Merulinidae, Montastraeidae, and Diploastraeidae (Cnidaria: Anthozoa: Scleractinia). Zool J Linn Soc 171: 277–355.

[57] Kitano YF, Benzoni F, Arrigoni R, Shirayama Y, Wallace CC and Fukami H (2014) A phylogeny of the family Poritidae (cnidaria, scleractinia) based on molecular and morphological analyses. PLoS One 9: e98406 10.1371/journal.pone.0098406 24871224 PMC4037213

[58] Zietara MS, Arndt A, Geets A, Hellemans B and Volckaert FA (2009) The nuclear rDNA region of *Gyrodactylus arcuatus* and *G*. *branchicus* (Monogenea: Gyrodactylidae). J Parasitol 86: 1368–1373. 10.1645/0022-3395(2000)086[1368:TNRROG]2.0.CO;2 11191921

[59] Collins AG, Winkelmann S, Hadrys H and Schierwater B (2005) Phylogeny of Capitata and Corynidae (Cnidaria, Hydrozoa) in light of mitochondrial 16S rDNA data. Zool Scr 34: 91–99.

[60] Nawrocki AM, Schuchert P and Cartwright P (2010) Phylogenetics and evolution of Capitata (Cnidaria: Hydrozoa), and the systematics of Corynidae. Zool Scr 39: 290–304.

[61] Schuchert P (2010) The European athecate hydroids and their medusae (Hydrozoa, Cnidaria): Capitata part 2. Rev Suisse Zool 117: 337–555.

[62] Nawrocki AM, Collins AG, Hirano YM, Schuchert P and Cartwright P (2013) Phylogenetic placement of *Hydra* and relationships within Aplanulata (Cnidaria: Hydrozoa). Mol Phylogenet Evol 67: 60–71. 10.1016/j.ympev.2012.12.016 23280366

[63] Folmer O, Black M, Hoeh W, Lutz R and Vrijenhoek R (1994) DNA primers for amplification of mitochondrial cytochrome c oxidase subunit I from diverse metazoan invertebrates. Mol Mar Biol Biotechnol 3: 294–299. 7881515

[64] Katoh K, Misawa K, Kuma Ki and Miyata T (2002) MAFFT: a novel method for rapid multiple sequence alignment based on fast Fourier transform. Nucleic Acids Res 30: 3059–3066. 12136088 10.1093/nar/gkf436 PMC135756

[65] Katoh K and Standley DM (2013) MAFFT multiple sequence alignment software version 7: improvements in performance and usability. Mol Biol Evol 30: 772–780. 10.1093/molbev/mst010 23329690 PMC3603318

[66] Tamura K, Stecher G, Peterson D, Filipski A and Kumar S (2013) MEGA6: Molecular Evolutionary Genetics Analysis version 6.0. Mol Biol Evol 30: 2725–2729. 10.1093/molbev/mst197 24132122 PMC3840312

[67] Swofford DL (2003) PAUP*. Phylogenetic analysis using parsimony (* and other methods) Version 4.b.10. Sunderland, MA: Sinauer Associates.

[68] Farris JS, Källersjö M, Kluge AG and Bult C (1995) Constructing a significance test for incongruence. Syst Biol 44: 570–572.

[69] Evans NM, Lindner A, Raikova EV, Collins AG and Cartwright P (2008) Phylogenetic placement of the enigmatic parasite, *Polypodium hydriforme*, within the Phylum Cnidaria. BMC Evol Biol 8: 139 10.1186/1471-2148-8-139 18471296 PMC2396633

[70] Cartwright P, Evans NM, Dunn CW, Marques AC, Miglietta MP, Schuchert P, et al (2008) Phylogenetics of Hydroidolina (Hydrozoa: Cnidaria). J Mar Biol Assoc U K 88: 1663–1672.

[71] Nylander J (2004) MrModeltest v2. Software distributed by the author Evolutionary Biology Centre, Uppsala University.

[72] Ronquist F and Huelsenbeck JP (2003) MrBayes 3: Bayesian phylogenetic inference under mixed models. Bioinformatics 19: 1572–1574. 12912839 10.1093/bioinformatics/btg180

[73] Drummond AJ and Rambaut A (2007) BEAST: Bayesian evolutionary analysis by sampling trees. BMC Evol Biol 7: 214 17996036 10.1186/1471-2148-7-214 PMC2247476

[74] Guindon S and Gascuel O (2003) A simple, fast and accurate algorithm to estimate large phylogenies by maximum likelihood. Syst Biol 52: 696–704. 14530136 10.1080/10635150390235520

[75] Rozas J, Sanchez-DelBarrio JC, Messeguer X and Rozas R (2003) DnaSP, DNA polymorphism analyses by the coalescent and other methods. Bioinformatics 19: 2496–2497. 14668244 10.1093/bioinformatics/btg359

[76] Bandelt HJ, Forster P and Röhl A (1999) Median-joining networks for inferring intraspecific phylogenies. Mol Biol Evol 16: 37–48. 10331250 10.1093/oxfordjournals.molbev.a026036

[77] Migotto AE (1996) Benthic shallow-water hydroids (Cnidaria, Hydrozoa) of the coast of São Sebastião, Brazil, including a checklist of Brazilian hydroids. Zool Verh Leiden 306: 1–125.

[78] Hirohito ES (1988) The hydroids of Sagami Bay. Tokyo: Publications of the Biolological Laboratory, Imperial Household.

[79] Schuchert P (2005) Species boundaries in the hydrozoan genus *Coryne* . Mol Phylogenet Evol 36: 194–199. 15904866 10.1016/j.ympev.2005.03.021

[80] Miglietta MP, Piraino S, Kubota S and Schuchert P (2007) Species in the genus *Turritopsis* (Cnidaria, Hydrozoa): a molecular evaluation. J Zool Syst Evol Res 45: 11–19.

[81] Folino-Rorem NC, Darling JA and D’Ausilio CA (2009) Genetic analysis reveals multiple cryptic invasive species of the hydrozoan genus *Cordylophora* . Biol Invasions 11: 1869–1882.

[82] Palumbi SR (1992) Marine speciation on a small planet. Trends Ecol Evol 7: 114–118. 10.1016/0169-5347(92)90144-Z 21235975

[83] Palumbi SR (1994) Genetic divergence, reproductive isolation, and marine speciation. Annu Rev Ecol Syst 25: 547–572.

[84] Martinez DE, Iniguez AR, Percell KM, Willner JB, Signorovitch J, et al (2010) Phylogeny and biogeography of *Hydra* (Cnidaria: Hydridae) using mitochondrial and nuclear DNA sequences. Mol Phylogenet Evol 57: 403–410. 10.1016/j.ympev.2010.06.016 20601008

[85] Lindner A, Govindarajan AF and Migotto AE (2011) Cryptic species, life cycles, and the phylogeny of *Clytia* (Cnidaria: Hydrozoa: Campanulariidae). Zootaxa: 23–36.

