# Supplementary figures and images for: The Hidden Diversity of Zanclea Associated with Scleractinians Revealed by Molecular Data
Source: PLoS One. 2015 Jul 24;10(7):e0133084. doi: 10.1371/journal.pone.0133084 (PMC4514839; doi:10.1371/journal.pone.0133084)

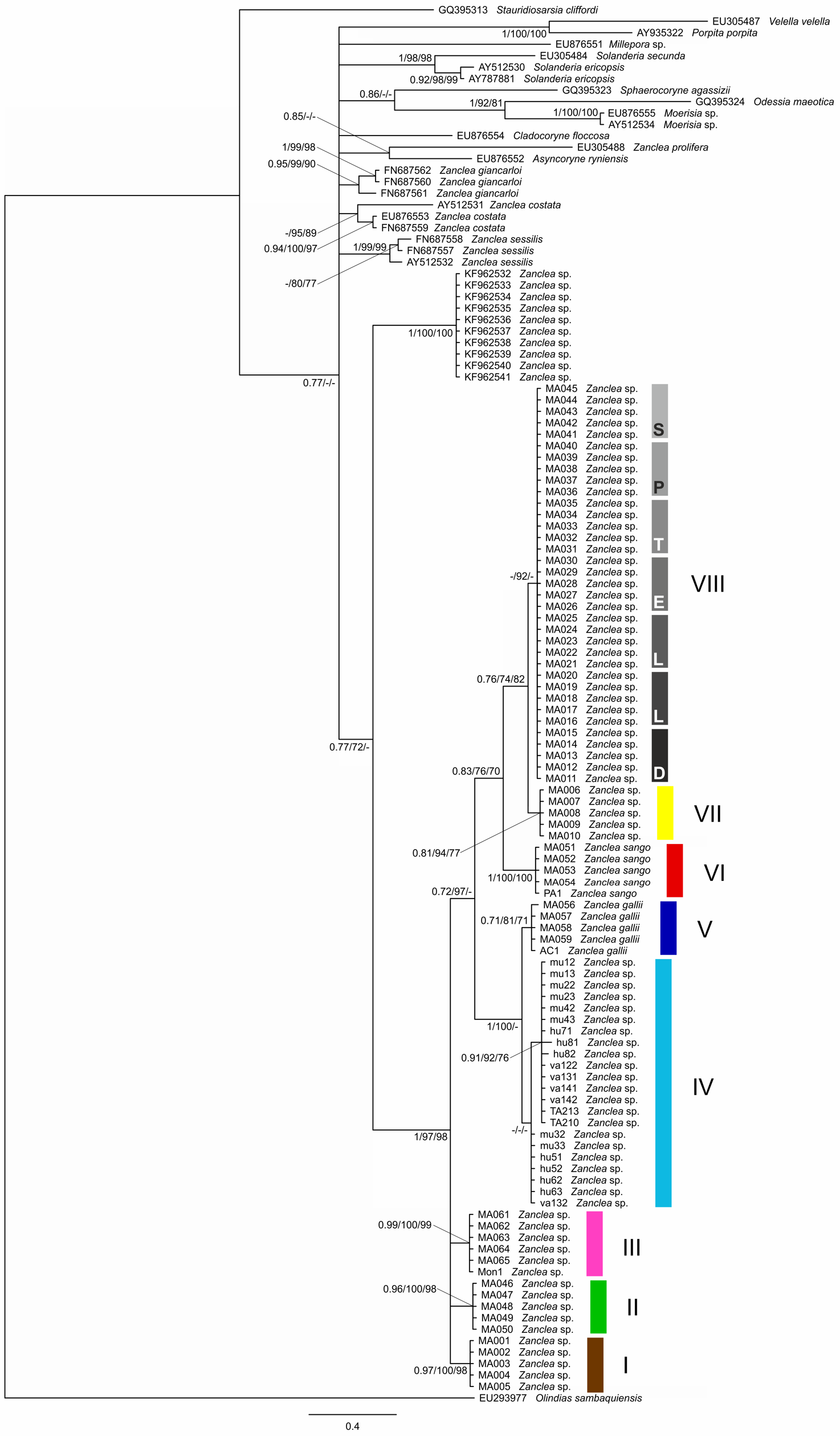

Supplement: S2 Fig — The clade support values are a posteriori probabilities (≥ 0.7), bootstrap values from Maximum Likelihood (≥ 70), and bootstrap values from Maximum Parsimony (≥ 70), in this order. (TIF) [file pone.0133084.s002.tif]

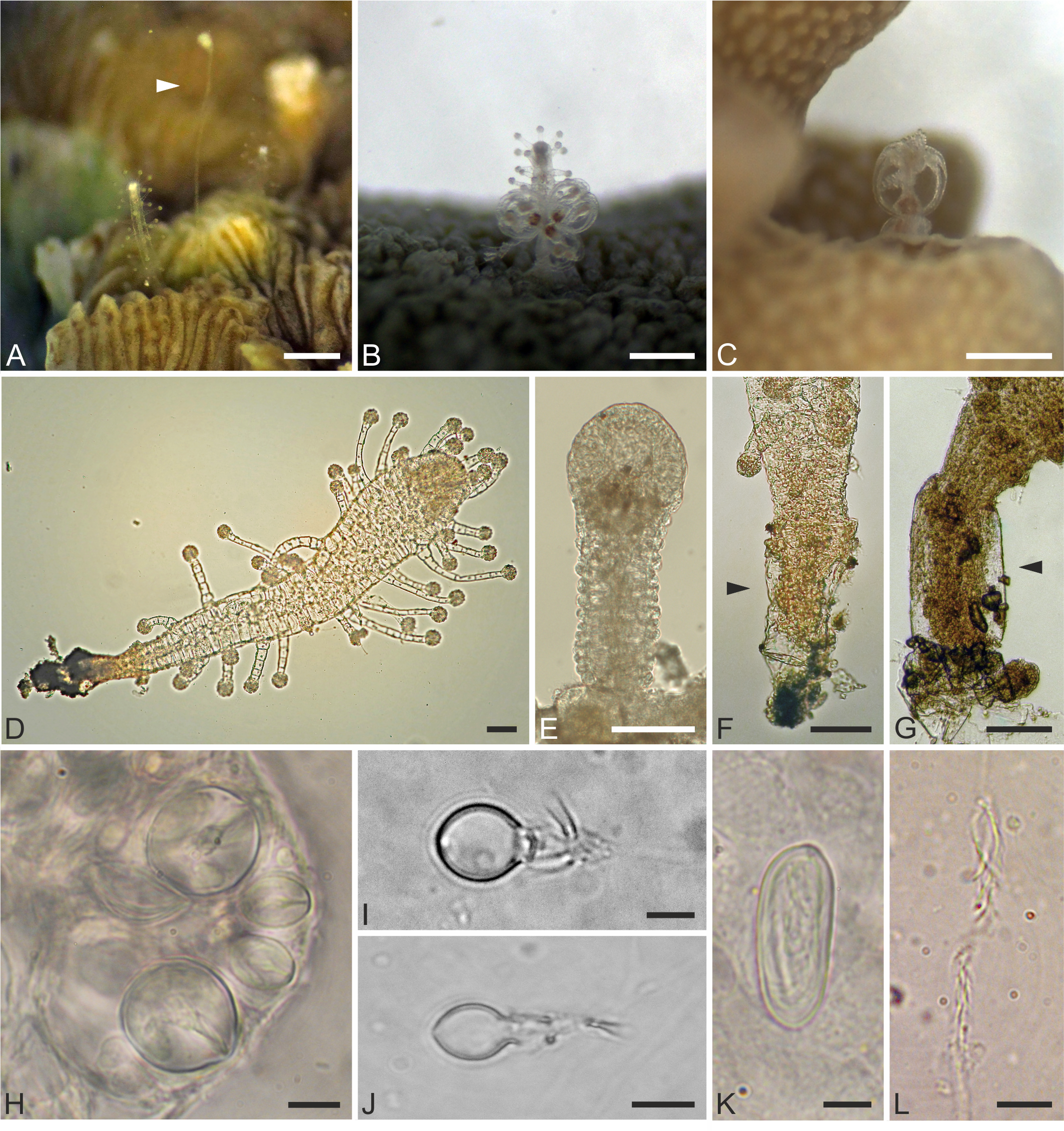

Supplement: S4 Fig — A) Gastrozooids and a dactylozooid (arrowhead) emerging from Pavona varians; B-C) Gastrogonozooid and a blastostyle bearing mature medusa buds on Porites sp. and Acropora muricata, respectively. D) An extended polyp belonging to clade VIII and growing on Turbinaria sp.; E) a contracted dactylozooid belonging to a Zanclea sango colony. F-G) Micrographs showing the basal portion of Zanclea hydroids associated with Leptoseris sp. and Leptastrea sp., respectively; the hydrocauli are covered by a transparent perisarc (arrowheads). H) Undischarged two-sized stenoteles; I-J) large and small discharged stenoteles. K-L) Undischarged apotrichous macrobasic eurytele from Zanclea sango and a detail of the distal part of the shaft of the same discharged nematocyst. (Scale bars: A-C ~ 0.5 mm; D-G ~ 100 μm; H-L ~ 5 μm). (TIFF) [file pone.0133084.s004.tiff]
